# The double-balloon technique: a safe and effective adjunctive technique in patients undergoing arterial therapy for hepatic malignancies with vascular supply not amenable to selective administration

**DOI:** 10.1186/s42155-023-00349-y

**Published:** 2023-02-07

**Authors:** Mari Tanaka, Raul Uppot, Dania Daye, Raymond Liu, Eric Wehrenberg-Klee

**Affiliations:** Division of Interventional Radiology, Department of Radiology, Massachusetts General Hospital, Harvard Medical School, Boston, MA USA

**Keywords:** Interventional oncology, Radioembolization, Chemoembolization

## Abstract

**Purpose:**

During catheter directed intraarterial therapy for liver lesions, challenging hepatic vascular anatomy can sometimes prevent selective administration of treatment delivery to liver tumors leading to increased toxicity to normal liver parenchyma. The objective of this study is to describe a variation of the double balloon technique that isolates the feeding artery to liver tumors proximally and distally to provide treatment delivery in lesions that cannot be otherwise selected.

**Materials and methods:**

An IRB-approved retrospective review of 7 patients who had undergone either radioembolization, chemoembolization, or bland embolization and the double balloon technique was employed. The devices used for flow augmentation were two 2.1 French balloon microcatheters (Sniper™, Embolx). One balloon was inflated distal to target vessel and the second was inflated proximal to protect from reflux.

**Results:**

DEB-TACE was performed in 3 cases, ^90^Y was performed in 4, and bland embolization was performed in the last patient. There were no adverse effects from the procedure or clinically evident effects from non-target embolization. Mean follow up time was 286.4 +/− 200.1 days. Six of the 7 patients are alive. One patient passed away on post-procedure day 121 from septic shock unrelated to the procedure. One patient was bridged to transplant with an additional TACE of a separate lesion.

**Conclusion:**

Double-balloon technique for patients undergoing ^90^Y or chemoembolization is a safe adjunctive technique for super selective treatment of hepatic lesions where direct selection via catheter is not feasible. This may increase the range of lesions that can be both safely and effectively treated by catheter directed therapies.

**Supplementary Information:**

The online version contains supplementary material available at 10.1186/s42155-023-00349-y.

## Introduction

Exposure of normal hepatic parenchyma to therapy during hepatic arterial embolization procedures can lead to liver toxicity especially in patients with cirrhosis or who have had chemotherapy (Lam et al., 2013; Gil-Alzugaray et al., 2013; Salem & Thurston, 2006). Super-selective administration of arterial therapy increases treatment delivery to the target lesion while simultaneously decreasing off-target hepatic toxicity, however challenging hepatic vascular anatomy, including feeder vessels too small to canulate with microcatheters, can sometimes prevent selective administration to tumor.

Flow redistribution with coils or other embolic material has been described to divert flow away from non-target tissue (Spreafico et al., 2015). Well established examples include using coils in the gastroduodenal or other visceral arteries to protect the gastrointestinal tract from non-target embolization. Intrahepatic use of plugs, coils, gelatin slurry, as well as balloons for distal protection or truncation followed by proximal delivery of radio embolic have been described as safe and effective in reducing non-target embolization (Core et al., 2020). Proximal delivery via anti-reflux microcatheters in addition to these techniques may further reduce non-target embolization.

Temporary occlusion of the distal angiosome using a balloon microcatheter to deliver ytriium-90 (^90^Y) has also been described as a safe and effective way to delivery radiation to target tissue while protecting non-target tissue in patients where the direct tumor-feeding vessel cannot be selected (Meek et al., 2019; Hagspiel et al., 2013). Hagspiel et al. described the use of balloons for temporary extrahepatic protection from non-target embolization during ^90^Y embolization (Hagspiel et al., 2013). Meek et al. described their experience with 17 cases where a single balloon microcatheter distally was used to temporarily redistribute flow in patients undergoing radioembolization of hepatic tumors (Meek et al., 2019).

While these maneuvers can spare distal hepatic parenchyma, they do not address issues of bead reflux, which can often be encountered within small treatment beds and lead to embolization of normal hepatic parenchyma or non-target gastrointestinal reflux. Recently, Soga et al. used two balloon microcatheters via a 4F Rosch hepatic catheter to perform TACE to a caudate lesion with vascular supply unable to be superselelected, with reduction in tumor size and resolution following additional treatments (Soga et al., 2020). We present a variation of the double-balloon technique (DBT) as a safe technique to protect both distal and proximal parenchyma from toxicity during treatment of lesions with difficult anatomy throughout the liver and for treatment to lesions with TACE, bland embolization, and radioembolization. This demonstrates the broad applicability of this technique to different liver directed therapy modalities and throughout the liver.

## Materials and methods

This retrospective, single-center study was conducted with institutional review board approval and written, informed patient consent.

### Study population

Between August 2020 and February 2022, seven patients (6 male, 1 female, age 57–85) in whom DBT was performed during catheter directed intra-arterial treatment of hepatic lesions. Patients were selected based on clinical history and imaging findings on planning MAA study and Dyna-CT. Those felt to benefit were 1) those with tumor anatomy such that selective embolization could not be achieved on planning MAA-angiogram and 2) desire to preserve liver parenchyma due to multiple reasons including hepatic function abnormalities, prior radioembolization, etc. Five of the 7 patients in this study had hepatocellular carcinoma (HCC), while one had metastatic neuroendocrine tumor (NET), and the other patient had metastatic uterine carcinoma. 5 of 7 had prior interventional radiology procedures such as prior catheter directed intra-arterial therapy and/or percutaneous ablation and 5 of 7 also or had additional interventional radiology procedures after the one where DBT was used. Patient demographics summarized in Table 1.

**Table 1:** Patient demographics

Case Number	Age (years)	Sex	Malignancy	Prior Interventional Radiology Procedure(s)
1	67	M	Hepatocellular	None
2	71	F	Metastatic uterine	MWA
3	71	M	HCC	TACE ×2, MWA
4	66	M	HCC	TACE, MWA, SIRT
5	57	M	HCC	None
6	78	M	Neuroendocrine	None
7	85	M	HCC	None

### Procedure technique

**Fig. 1:**
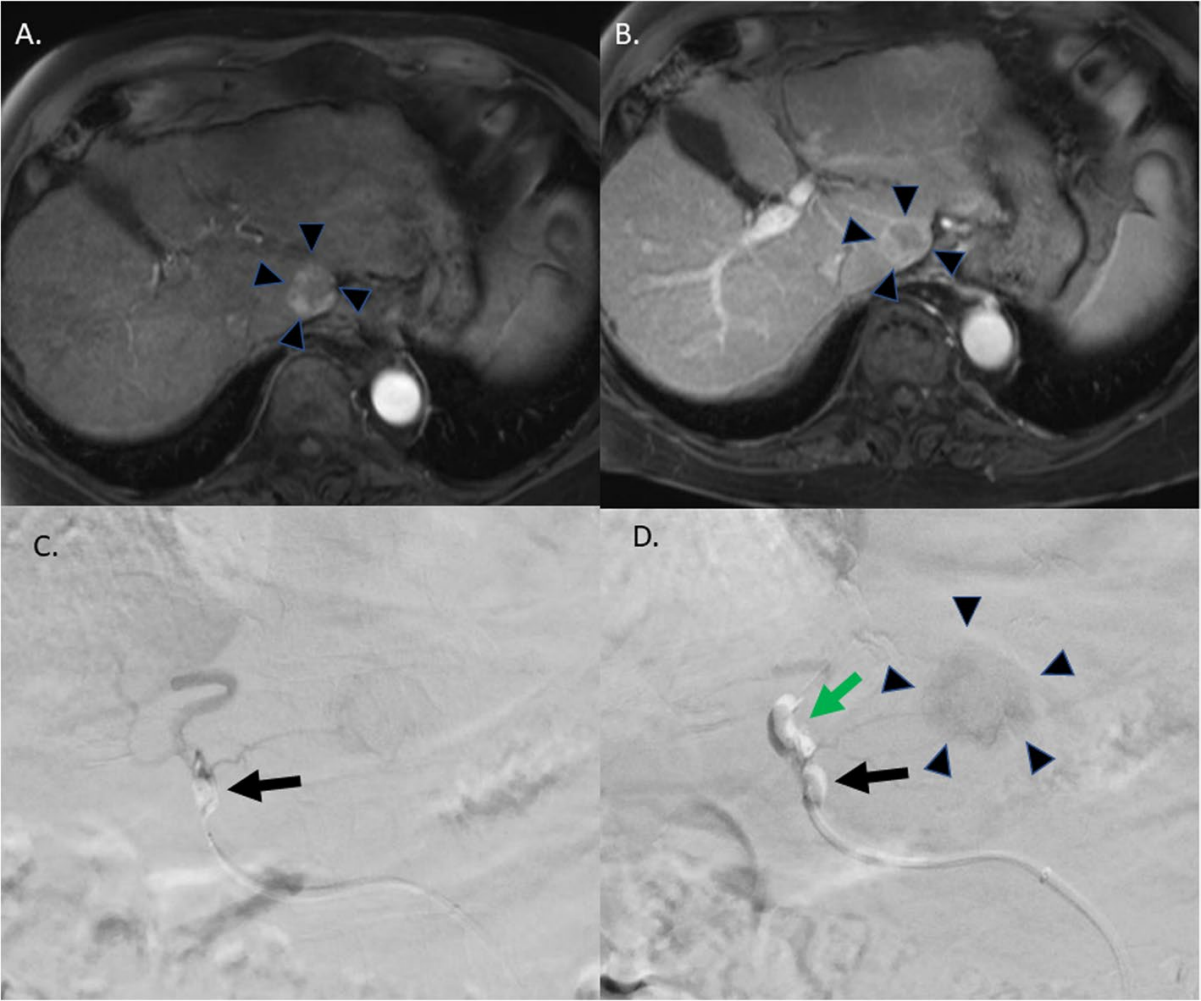
**a-d** Treatment of a OPTN5B caudate lesion. **a** Surveillance MRI in this patient with cirrhosis demonstrated an arterially enhancing lesion measuring 2.3 cm centered in segment 1(black arrow heads) with **b** early washout and pseudocapsule formation deeming it OPTN5b and was referred for Y-90 radioembolization. His Pre-SIRT and SIRT studies demonstrated the caudate lobe artery arising from the proximal left hepatic artery with single tumor blush but with substantial adjacent supply to segments II/III and IV. Given desire to reduce risk of hepatic parenchymal embolization due to comorbidities of cirrhosis and portal hypertension, the double balloon technique was used. A 2.1 French Sniper catheter was positioned beyond the caudate artery. **c** A second 2.1 French angled Sniper catheter was placed and positioned at the proximal left hepatic artery proximal to the caudate artery (black arrow). **d** Both balloons were inflated with dilute contrast per manufacturer protocol (green and black arrows). Digital subtraction angiography from the proximal catheter demonstrated selective flow of contrast into caudate artery with avid enhancement of target lesion (black arrow heads), and minimal, sluggish flow beyond the second/distal Sniper balloon, so Y-90 Theraspheres were administered

**Fig. 2:**
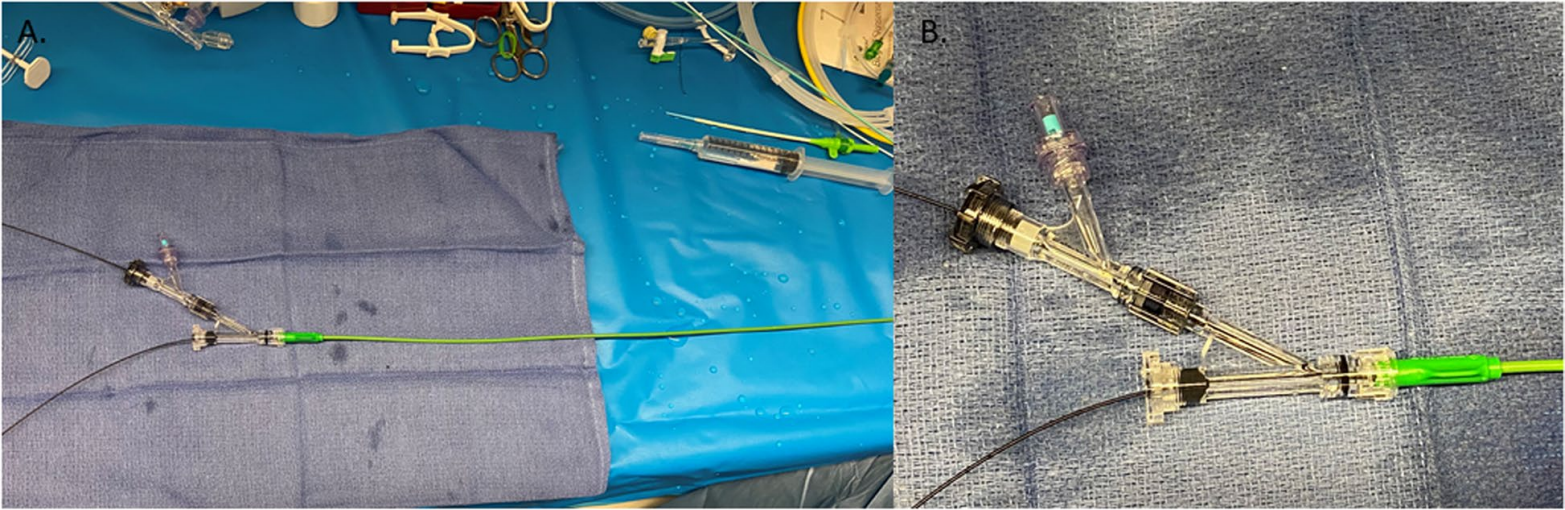
**a** Set up for double balloon technique**.** The hemostatic valve of a Destination sheath is removed and replaced with a rotating hemostatic valve. The microcatheter is introduced into the vascular sheath and a rotating hemostatic valve is used. **b** Through the flush port of the rotating hemostatic valve a second rotating hemostatic valve is attached which allows for the introduction of the second microcatheter

Diagnostic cross-sectional imaging was reviewed in a multidisciplinary manner prior to patient being booked for interventional radiology procedures. Lesions were identified on malignancy follow-up or surveillance imaging such as abdominal MRI liver protocol to identify HCC lesions (Fig. 1a, b). Coagulation studies as well as platelet counts were obtained within 7 days prior to the procedure according to institutional protocol. Anticoagulation medications were stopped as per institutional protocol. Sedation was provided by either radiology nursing or the department of anesthesia using midazolam, fentanyl, and propofol if deemed appropriate by the anesthesia team. Anti-coagulation or heparin is not routinely administered during the procedure in our practice. Interventions were performed by three interventional radiologists with 5–15 years of experience in interventional oncology procedures. Arterial access was achieved via right common femoral artery access using a micropuncture set and access upsized to a 6F × 45 cm Destination sheath (Terumo). The celiac and/or superior mesenteric artery (SMA) were selected with a 5F × 65 cm C2 catheter over an 0.035 wire and positioned well within the hepatic artery to secure access. The 6Fr sheath was then advanced over the C2 catheter until the sheath was seated within the common hepatic artery. Cone-beam CT was performed at time of treatment or during prior mapping procedure to delineate exact takeoff of target vessel. At this point the C2 catheter was withdrawn and the hemostatic valve removed. The hemostatic valve is then replaced with sequential rotating hemostatic valves (RHV). Through each RHV, a single 2.1 French balloon microcatheter (Sniper™, Embolx) is placed (Fig. 2). The first balloon microcatheter was advanced distal to the feeding vessel. Proper positioning was confirmed with digital subtraction angiography (DSA). A second balloon occlusion microcatheter was then placed into the 6F sheath adjacent to the previous microcatheter and advanced proximal to the origin of the target vessel feeding the tumor and proper location was confirmed using DSA (Fig. 1c). Both microcatheter balloons were inflated and if subsequent DSA from the proximal microcatheter demonstrated avid enhancement of the tumor (Fig. 1d), then delivery of treatment (doxorubicin loaded beads, ^90^Y, or bland Embospheres) was performed. Thrombosis is not associated with the balloon inflated microcatheters, so we do not routinely anti-coagulation patients or run continuous flush through the microcatheters.

### Technique variations

#### Case 1

**Fig. 3:**
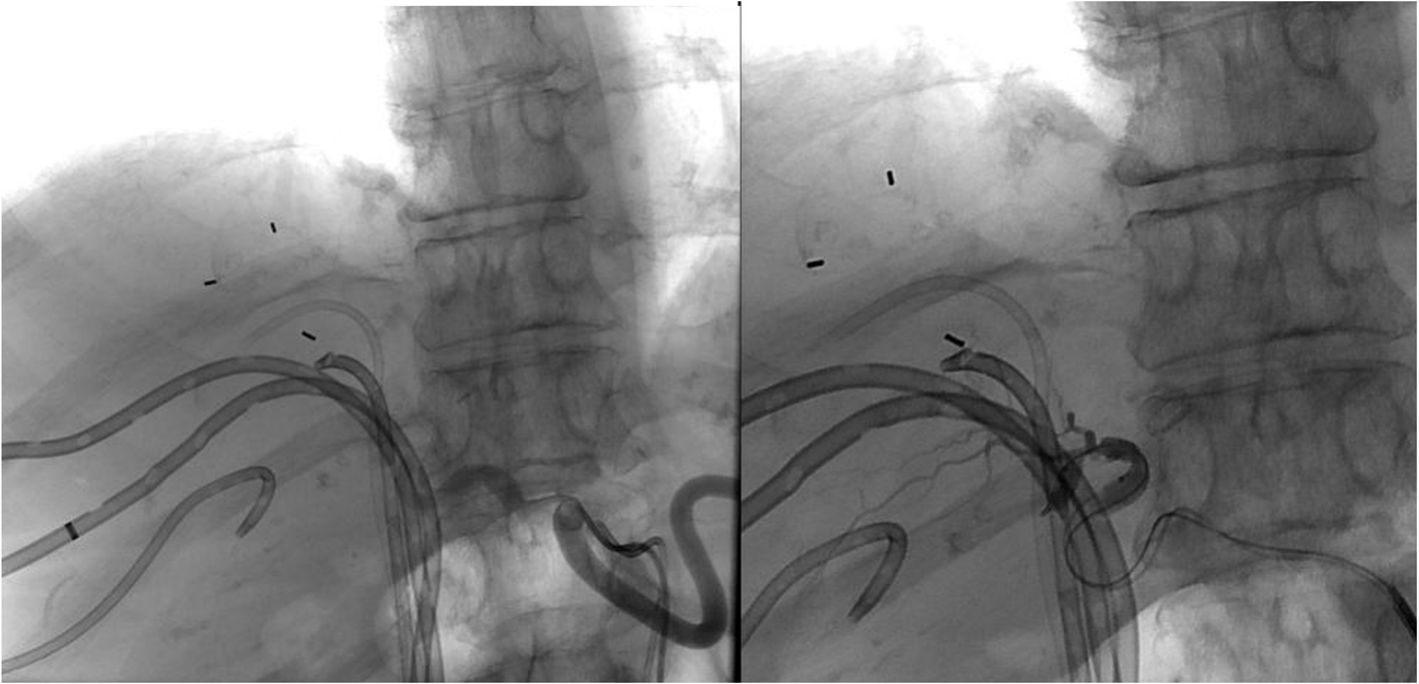
**a**, **b** Variation of DBT using two Simmons 1 catheters. **a** This patient had a stenotic and downward going celiac axis that would not allow for the destination sheath to track, so via bilateral common femoral access two 5F Simmons 1 catheters were used to select the celiac axis (black and white arrow heads). **b** Through each of these 5F catheters the 2.1 French sniper catheters were positioned, balloons inflated (green arrows), and the lesion was treated

Patients may not have vascular anatomy that allows for sheath to track. A patient with multiple prior biliary drainage catheters referred for treatment had downward oriented celiac access with ostial stenosis and efforts to track a Destination sheath were unsuccessful. To facilitate the DBT technique, contralateral left common femoral access was obtained due to operator preference, but radial access could also be considered. Two Simmons 1 catheters were then used to select the celiac access (Fig. 3a). Through these parent catheters the Sniper microcatheters were placed and allowed for successful DBT and treatment of lesion (Fig. 3b).

#### Case 2

**Fig. 4:**
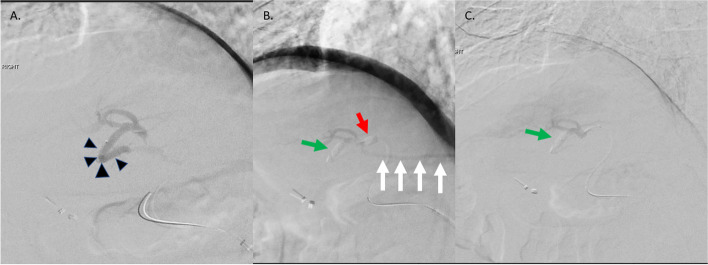
**a-c** Variation of DBT with unexpected flow redistribution. **a** An angiogram from the left hepatic artery demonstrated contributing vessels to target lesion in segment 4 with adjacent origin of the segment 3 arteries (black arrow head). **b** A Sniper microcatheter was placed in to the segment 3 arteries and balloon inflated to protect segment 3 parenchyma (green arrow), and a second microcatheter placed proximal to direct flow into feeding branches (red arrow). However, flow to segment 2 was identified (white arrows). **c** Interestingly, when the proximal balloon was deflated, less flow was noted to segment 2, so this area was treated with only distal occlusion

We encountered a situation where to treat a lesion in segment 4 for which the vascular supply arose from the left hepatic artery. Arterial branches to segment 2 and 3 arose in close proximity and did not supply the tumor (Fig. 4a). The DBT was employed to isolate the two segment 4 vessels thought to supply the lesion. Distally, a Sniper microcatheter was advanced to protect segment 3 and proximal to the origin of the two feeding vessels a second Sniper microcatheter was advanced. Interestingly when the proximal balloon was inflated, there was more flow towards segment 2 than when deflated (Fig. 4b and c). Because of this, decision was made to treat with distal balloon up and proximal balloon down.

#### Case 3

In tall patients or when radial access is used, it may be necessary to use a slightly longer Destination sheath to secure access into the celiac artery. In such cases a 6Fr Terumo R2P sheath can be used along with 150 cm balloon microcatheters.

## Results

All patients tolerated the procedures well without immediate complications and there were no adverse effects from the procedure or clinically evident effects from non-target embolization. Patients who underwent trans-arterial chemoembolization (TACE) or bland embolization were admitted for pain control and were discharged on post-procedure day 1 or 2. The mean size of the hepatic lesion treated was 4.6 **±** 2.4 cm. The size of target, hepatic segment location, and embolization material used are summarized in Table 2.

**Table 2:** Technical details about embolization material, and lesion size/location

Case Number	Lesion Size (cm)	Lesion Location	DynaCT Performed?	Embolic Material (dose)
1	2.6 cm	segment 1	Yes	Theraspheres
2	9.2 cm	segment 6/7	No	Theraspheres
3	1.6 cm	segment 4b	Yes	100-300um DC beads loaded with doxorubicin
4	6.3 cm	segment 6	No	100-300u DC beads loaded with doxorubicin
5	5.0 cm	Segment 1	No	100-300u DC beads loaded with doxorubicin
6	2.6 cm	segment 1	No	300-500um Embospheres
7	6.3 cm, 2.5 cm	Segment 4/8	Yes	Theraspheres

One patient passed away on post-procedure day 121 from septic shock without source identified. The remaining 6 patients are alive with mean follow up of 358.4 **±** 175.1 days. One patient successfully bridged to transplant following an additional TACE procedure. Five of the patients underwent additional subsequent procedures with interventional radiology. Across all patients, no significant changes in AST/ALT levels pre- and one- month post-intervention were observed suggesting no significant hepatic parenchymal involvement (Supplemental Table 1). In four patients with HCC there were no changes in Child Pugh scores or albumin bilirubin gradient scores following intervention.

## Discussion

The double balloon technique is safe for targeted delivery of treatment in difficult vascular anatomy and in patients where sparing of normal parenchyma is paramount. All patients in this study had either previously undergone liver-directed procedures for their disease and/or underwent additional procedures to address other sites of disease, which highlights the importance of protecting normal liver parenchyma as much as possible to preserve liver function.

The DBT has limitations: the first is increased procedure time and cost due to increased use of hardware. There is a theoretical increased risk of proximal thrombosis but this has not been associated with balloon inflated microcatheters. In addition to challenges of proximal celiac artery anatomy that may be overcome either through the radial or through a double parent catheter variation, there are additional potential limitations to the use of DBT. The use of the 6F vascular sheath may cause injury when negotiated into the common hepatic artery, and if not feasible variations of this technique can be performed, as described in Case 1. As highlighted in Case 2, the hepatic arteries are not terminal arterioles, and thus intra-hepatic arterial-arterial shunts may be seen in the context of proximal hepatic arterial branch balloon occlusion. If this is seen intraprocedurally, alternative angiosomal truncation methods, such a gel-foam embolization may be pursued. Additionally, application in large hepatic arteries may be limited by the Sniper balloon maximum inflation diameter of 6 mm.

## Conclusion

Double-balloon technique for in patients undergoing ^90^Y or chemoembolization is a safe adjunctive technique for super selective treatment of hepatic lesions where direct selection via catheter is not feasible. This may increase the range of lesions that can be both safely and effectively treated by catheter directed therapies.

## Supplementary Information

**Additional file 1: Supplemental Table 1.** Changes in AST and ALT pre- and post-DBT intervention.

## Data Availability

The datasets generated and/or analyzed during the current study are not publicly available due to institution data privacy policy, but are available from the corresponding author on reasonable request.

## Abbreviations

DBT: Double balloon technique
DSA: Digital subtraction angiography
HCC: Hepatocellular carcinoma
NET: Neuroendocrine tumor
SMA: Superior mesenteric artery
TACE: Trans-arterial chemoembolization
^90^Y: Yttrium-90
