# Supplementary material for: The double-balloon technique: a safe and effective adjunctive technique in patients undergoing arterial therapy for hepatic malignancies with vascular supply not amenable to selective administration
Source: CVIR Endovasc. 2023 Feb 7;6:3. doi: 10.1186/s42155-023-00349-y (PMC9902581; doi:10.1186/s42155-023-00349-y)
Supplement: Supplementary file 1 — Additional file 1: Supplemental Table 1. Changes in AST and ALT pre- and post-DBT intervention. [file 42155_2023_349_MOESM1_ESM.docx]

Supplemental Table 1: Changes in AST and ALT pre- and post-DBT intervention

| **Case Number** | **AST/ALT**  **Pre-intervention**  **(U/L)** | **AST/ALT**  **Post -intervention**  **(U/L)** |  |
| --- | --- | --- | --- |
| 1 | 48/27 | 22/9 |  |
| 2 | 23/36 | 46/20 |  |
| 3 | 59/28 | 59/28 |  |
| 4 | 62/35 | 57/31 |  |
| 5 | 26/20 | 25/20 |  |
| 6 | 33/30 | 22/20 |  |
| 7 | 37/31 | 39/30 |  |
| **Mean +/- standard deviation** | AST: 41.1 +/- 15  ALT: 29.6 +/- 5.4 | AST: 38.6 +/-16.1  ALT: 22.6 +/- 7.7 | AST: p = 0.421  ALT: p = 0.053 |
